# Diethyl 1,4-dioxo-1,2,2a,3,4,10b-hexahydro-5*H*,10*H*-2,3,4a,10a-tetraaza­benzo[*g*]cyclopenta[*cd*]azulene-2a,10b-dicarboxylate

**DOI:** 10.1107/S1600536809014548

**Published:** 2009-04-25

**Authors:** Jing Qin

**Affiliations:** a Key Laboratory of Pesticides and Chemical Biology of the Ministry of Education, College of Chemistry, Central China Normal University, Wuhan 430079, People’s Republic of China

## Abstract

In the title compound, C_18_H_20_N_4_O_6_, the dihedral angle between the two fused five-membered rings in the glycoluril unit is 64.42 (2)°. The crystal structure features inter­molecular N—H⋯O and C—H⋯O interactions.  An intramolecular C—H⋯O contact is also present.

## Related literature

For the preparation of the title compound, see: Wu *et al.* (2002*a* ▶). For crystal engineering studies of glycoluril and its derivatives, see: Chen *et al.* (2007 ▶); Wang *et al.* (2006 ▶); Johnson *et al.* (2002 ▶); Wu *et al.* (2002*b* ▶).
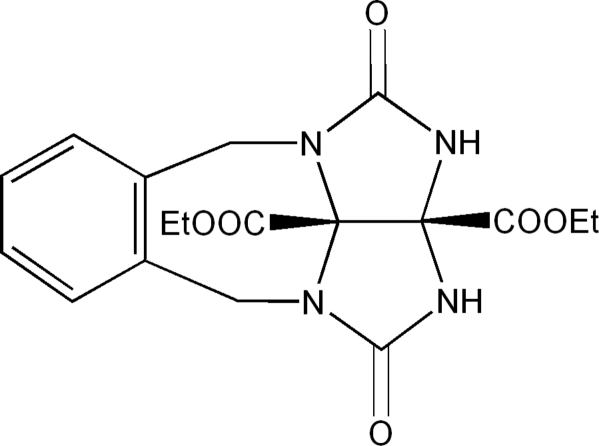


## Experimental

### 

#### Crystal data

C_18_H_20_N_4_O_6_
*M* *_r_* = 388.38
Triclinic, 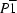

*a* = 8.1394 (5) Å
*b* = 9.4425 (5) Å
*c* = 13.3576 (8) Å
α = 93.1550 (10)°
β = 96.0560 (10)°
γ = 112.3970 (10)°
*V* = 938.80 (9) Å^3^
*Z* = 2
Mo *K*α radiation
μ = 0.11 mm^−1^
*T* = 294 K
0.20 × 0.20 × 0.10 mm

#### Data collection

Bruker SMART 4K CCD area-detector diffractometer
Absorption correction: multi-scan (*SADABS*; Sheldrick, 1997 ▶) *T*_min_ = 0.976, *T*_max_ = 0.989
7700 measured reflections
3624 independent reflections
3028 reflections with *I* > 2σ(*I*)
*R*_int_ = 0.020

#### Refinement

*R*[*F* ^2^ > 2σ(*F* ^2^)] = 0.049
*wR*(*F* ^2^) = 0.144
*S* = 1.05
3624 reflections
261 parameters
H atoms treated by a mixture of independent and constrained refinement
Δρ_max_ = 0.28 e Å^−3^
Δρ_min_ = −0.20 e Å^−3^

### 

Data collection: *SMART* (Bruker, 2001 ▶); cell refinement: *SAINT* (Bruker, 2001 ▶); data reduction: *SAINT*; program(s) used to solve structure: *SHELXS97* (Sheldrick, 2008 ▶); program(s) used to refine structure: *SHELXL97* (Sheldrick, 2008 ▶); molecular graphics: *SHELXTL* (Sheldrick, 2008 ▶); software used to prepare material for publication: *SHELXTL*.

## Supplementary Material

Crystal structure: contains datablocks I, global. DOI: 10.1107/S1600536809014548/nc2142sup1.cif

Structure factors: contains datablocks I. DOI: 10.1107/S1600536809014548/nc2142Isup2.hkl

Additional supplementary materials:  crystallographic information; 3D view; checkCIF report

## Figures and Tables

**Table 1:** Hydrogen-bond geometry (Å, °)

*D*—H⋯*A*	*D*—H	H⋯*A*	*D*⋯*A*	*D*—H⋯*A*
C7—H7*A*⋯O3	0.97	2.52	3.107 (2)	119
C17—H17*B*⋯O2^i^	0.97	2.58	3.343 (3)	136
C7—H7*B*⋯O3^ii^	0.97	2.59	3.478 (2)	153
N4—H4*A*⋯O2^iii^	0.85 (3)	2.05 (3)	2.871 (2)	165 (2)
N3—H3*A*⋯O5^iv^	0.87 (3)	2.09 (3)	2.917 (2)	159 (2)

Symmetry codes: (i) 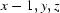; (ii) 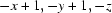; (iii) 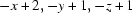; (iv) 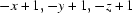.

**Fig. 1.**
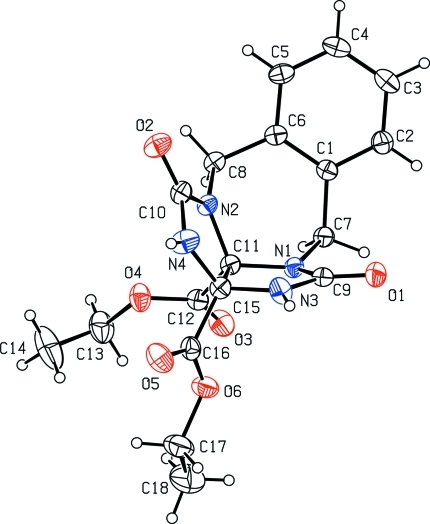
A view of (I), showing the atom-labelling scheme, with displacement ellipsoids drawn at the 30% probability level.

## supplementary crystallographic information

### Comment

Glycoluril and its derivatives have been widely studied in supramolecular chemistry (Johnson *et al.*, 2002; Wu *et al.*, 2002b). As a continuation of our recent studies in this area (Wang *et al.*, 2006; Chen *et al.*, 2007), we herein report the crystal structure of the title compound (Fig. 1). The dihedral angle between the two fused five-membered rings in the glycoluril unit is 64.42 (2) °. In the crystal structure the molecules are connected via weak intermolecular N—H···O hydrogen bonding (Table 1).

### Experimental

The title compound was synthesized according to a literature procedure (Wu *et al.*; 2002a). Crystals of (I) suitable for X-ray diffraction were grown by slow evaporation of a dichloromethane-methanol (1:2) solution of the title compound at room temperature.

### Refinement

All H atoms were positioned with idealized geometry with C—H = 0.93–0.97 Å (methyl H atoms allowed to rotate but not to tip) and were refined isotropic (*U*_iso_(H) = 1.2 *U*_eq_(C) or 1.5 *U*_eq_(C) (methyl C)) using a riding model.

### Crystal data

**Table:**

C_18_H_20_N_4_O_6_	*Z* = 2
*M**_r_* = 388.38	*F*(000) = 408
Triclinic, *P*1	*D*_x_ = 1.374 Mg m^−^^3^
Hall symbol: -P 1	Mo *K*α radiation, λ = 0.71073 Å
*a* = 8.1394 (5) Å	Cell parameters from 3262 reflections
*b* = 9.4425 (5) Å	θ = 2.4–27.5°
*c* = 13.3576 (8) Å	µ = 0.11 mm^−^^1^
α = 93.155 (1)°	*T* = 294 K
β = 96.056 (1)°	Block, colorless
γ = 112.397 (1)°	0.20 × 0.20 × 0.10 mm
*V* = 938.80 (9) Å^3^	

### Data collection

**Table:**

Bruker SMART 4K CCD area-detector diffractometer	3624 independent reflections
Radiation source: fine-focus sealed tube	3028 reflections with *I* > 2σ(*I*)
graphite	*R*_int_ = 0.020
φ and ω scans	θ_max_ = 26.0°, θ_min_ = 1.5°
Absorption correction: multi-scan (*SADABS*; Sheldrick, 1997)	*h* = −10→8
*T*_min_ = 0.976, *T*_max_ = 0.989	*k* = −11→11
7700 measured reflections	*l* = −16→16

### Refinement

**Table:**

Refinement on *F*^2^	Primary atom site location: structure-invariant direct methods
Least-squares matrix: full	Secondary atom site location: difference Fourier map
*R*[*F*^2^ > 2σ(*F*^2^)] = 0.049	Hydrogen site location: inferred from neighbouring sites
*wR*(*F*^2^) = 0.144	H atoms treated by a mixture of independent and constrained refinement
*S* = 1.05	*w* = 1/[σ^2^(*F*_o_^2^) + (0.0741*P*)^2^ + 0.3055*P*] where *P* = (*F*_o_^2^ + 2*F*_c_^2^)/3
3624 reflections	(Δ/σ)_max_ = 0.005
261 parameters	Δρ_max_ = 0.28 e Å^−^^3^
0 restraints	Δρ_min_ = −0.20 e Å^−^^3^

### Special details

**Table:**

Geometry. All e.s.d.'s (except the e.s.d. in the dihedral angle between two l.s. planes) are estimated using the full covariance matrix. The cell e.s.d.'s are taken into account individually in the estimation of e.s.d.'s in distances, angles and torsion angles; correlations between e.s.d.'s in cell parameters are only used when they are defined by crystal symmetry. An approximate (isotropic) treatment of cell e.s.d.'s is used for estimating e.s.d.'s involving l.s. planes.
Refinement. Refinement of *F*^2^ against ALL reflections. The weighted *R*-factor *wR* and goodness of fit *S* are based on *F*^2^, conventional *R*-factors *R* are based on *F*, with *F* set to zero for negative *F*^2^. The threshold expression of *F*^2^ > σ(*F*^2^) is used only for calculating *R*-factors(gt) *etc*. and is not relevant to the choice of reflections for refinement. *R*-factors based on *F*^2^ are statistically about twice as large as those based on *F*, and *R*- factors based on ALL data will be even larger.

### Fractional atomic coordinates and isotropic or equivalent isotropic displacement parameters (Å^2^)

**Table:**

	*x*	*y*	*z*	*U*_iso_*/*U*_eq_	
C1	0.7535 (3)	0.3332 (2)	0.06082 (12)	0.0350 (4)	
C2	0.6905 (3)	0.1940 (2)	0.00001 (15)	0.0442 (5)	
H2	0.5714	0.1512	−0.0298	0.053*	
C3	0.8025 (3)	0.1180 (2)	−0.01692 (16)	0.0516 (5)	
H3	0.7589	0.0250	−0.0579	0.062*	
C4	0.9787 (3)	0.1811 (2)	0.02733 (16)	0.0515 (5)	
H4	1.0552	0.1318	0.0152	0.062*	
C5	1.0421 (3)	0.3177 (2)	0.08982 (15)	0.0437 (5)	
H5	1.1607	0.3581	0.1207	0.052*	
C6	0.9321 (2)	0.3955 (2)	0.10733 (12)	0.0352 (4)	
C7	0.6302 (3)	0.4178 (2)	0.07030 (12)	0.0375 (4)	
H7A	0.6781	0.5130	0.0391	0.045*	
H7B	0.5141	0.3556	0.0321	0.045*	
C8	1.0095 (2)	0.5470 (2)	0.17322 (13)	0.0359 (4)	
H8A	1.1323	0.5666	0.2006	0.043*	
H8B	1.0123	0.6287	0.1316	0.043*	
C9	0.4886 (3)	0.3441 (2)	0.22434 (14)	0.0392 (4)	
C10	0.9584 (3)	0.5307 (2)	0.35329 (13)	0.0395 (4)	
C11	0.7426 (2)	0.5735 (2)	0.24235 (12)	0.0330 (4)	
C12	0.7751 (3)	0.7358 (2)	0.20982 (13)	0.0372 (4)	
C13	0.9500 (4)	1.0015 (3)	0.2570 (2)	0.0691 (7)	
H13A	1.0481	1.0306	0.2168	0.083*	
H13B	0.8494	1.0152	0.2195	0.083*	
C14	1.0045 (7)	1.0966 (3)	0.3520 (3)	0.1181 (14)	
H14A	0.9041	1.0729	0.3891	0.177*	
H14B	1.0469	1.2031	0.3398	0.177*	
H14C	1.0988	1.0774	0.3906	0.177*	
C15	0.6771 (3)	0.5423 (2)	0.34897 (13)	0.0379 (4)	
C16	0.6154 (3)	0.6578 (2)	0.40178 (14)	0.0412 (4)	
C17	0.4679 (4)	0.8288 (3)	0.3831 (2)	0.0741 (8)	
H17A	0.5609	0.9012	0.4335	0.089*	
H17B	0.3639	0.7756	0.4160	0.089*	
C18	0.4201 (6)	0.9114 (4)	0.3022 (3)	0.1120 (13)	
H18A	0.5252	0.9687	0.2727	0.168*	
H18B	0.3718	0.9808	0.3300	0.168*	
H18C	0.3321	0.8386	0.2511	0.168*	
N1	0.6035 (2)	0.45511 (17)	0.17377 (10)	0.0356 (4)	
N2	0.9101 (2)	0.55255 (17)	0.25637 (10)	0.0345 (4)	
N3	0.5268 (3)	0.3968 (2)	0.32473 (13)	0.0491 (4)	
H3A	0.456 (3)	0.355 (3)	0.3683 (19)	0.059*	
N4	0.8307 (3)	0.5358 (2)	0.40898 (13)	0.0532 (5)	
H4A	0.835 (3)	0.525 (3)	0.472 (2)	0.064*	
O1	0.37312 (19)	0.22369 (16)	0.18597 (12)	0.0528 (4)	
O2	1.0959 (2)	0.51339 (19)	0.38374 (10)	0.0531 (4)	
O3	0.7018 (2)	0.75960 (17)	0.13482 (10)	0.0552 (4)	
O4	0.8984 (2)	0.84003 (15)	0.27670 (11)	0.0526 (4)	
O5	0.6376 (3)	0.68292 (19)	0.49151 (11)	0.0644 (5)	
O6	0.5323 (2)	0.71765 (18)	0.33836 (11)	0.0556 (4)	

### Atomic displacement parameters (Å^2^)

**Table:**

	*U*^11^	*U*^22^	*U*^33^	*U*^12^	*U*^13^	*U*^23^
C1	0.0430 (10)	0.0384 (9)	0.0271 (8)	0.0191 (8)	0.0058 (7)	0.0050 (7)
C2	0.0461 (11)	0.0437 (10)	0.0410 (10)	0.0161 (9)	0.0054 (8)	0.0024 (8)
C3	0.0708 (15)	0.0391 (11)	0.0507 (12)	0.0266 (11)	0.0138 (11)	0.0024 (9)
C4	0.0657 (15)	0.0492 (12)	0.0557 (12)	0.0368 (11)	0.0181 (11)	0.0113 (10)
C5	0.0426 (11)	0.0528 (11)	0.0450 (10)	0.0261 (10)	0.0106 (9)	0.0166 (9)
C6	0.0391 (10)	0.0427 (10)	0.0286 (8)	0.0197 (8)	0.0072 (7)	0.0108 (7)
C7	0.0406 (10)	0.0476 (10)	0.0266 (8)	0.0221 (9)	−0.0021 (7)	−0.0009 (7)
C8	0.0340 (10)	0.0443 (10)	0.0305 (9)	0.0155 (8)	0.0059 (7)	0.0078 (7)
C9	0.0373 (10)	0.0384 (10)	0.0451 (10)	0.0186 (9)	0.0059 (8)	0.0022 (8)
C10	0.0412 (11)	0.0464 (10)	0.0293 (9)	0.0160 (9)	0.0003 (8)	0.0062 (8)
C11	0.0354 (10)	0.0382 (9)	0.0262 (8)	0.0151 (8)	0.0042 (7)	0.0044 (7)
C12	0.0419 (10)	0.0411 (10)	0.0312 (9)	0.0186 (9)	0.0061 (8)	0.0055 (7)
C13	0.0793 (18)	0.0386 (12)	0.0763 (17)	0.0093 (12)	0.0038 (14)	0.0123 (11)
C14	0.170 (4)	0.0489 (16)	0.107 (3)	0.019 (2)	0.001 (3)	−0.0135 (16)
C15	0.0405 (10)	0.0436 (10)	0.0296 (9)	0.0160 (9)	0.0055 (7)	0.0056 (7)
C16	0.0412 (11)	0.0415 (10)	0.0350 (10)	0.0092 (9)	0.0087 (8)	−0.0008 (8)
C17	0.0743 (18)	0.0650 (16)	0.096 (2)	0.0388 (15)	0.0268 (15)	−0.0014 (14)
C18	0.123 (3)	0.088 (2)	0.143 (3)	0.070 (2)	−0.014 (3)	0.001 (2)
N1	0.0346 (8)	0.0415 (8)	0.0296 (7)	0.0153 (7)	0.0002 (6)	0.0005 (6)
N2	0.0339 (8)	0.0433 (8)	0.0263 (7)	0.0155 (7)	0.0013 (6)	0.0051 (6)
N3	0.0571 (11)	0.0412 (9)	0.0423 (9)	0.0088 (8)	0.0185 (8)	0.0041 (7)
N4	0.0579 (12)	0.0867 (14)	0.0265 (8)	0.0393 (11)	0.0068 (8)	0.0152 (8)
O1	0.0427 (8)	0.0437 (8)	0.0624 (9)	0.0079 (7)	0.0054 (7)	−0.0045 (7)
O2	0.0493 (9)	0.0791 (11)	0.0373 (7)	0.0323 (8)	0.0003 (6)	0.0148 (7)
O3	0.0773 (11)	0.0517 (9)	0.0407 (8)	0.0328 (8)	−0.0054 (7)	0.0069 (6)
O4	0.0588 (9)	0.0372 (7)	0.0505 (8)	0.0091 (7)	−0.0048 (7)	0.0069 (6)
O5	0.0891 (13)	0.0677 (10)	0.0367 (8)	0.0307 (9)	0.0136 (8)	−0.0042 (7)
O6	0.0633 (10)	0.0630 (9)	0.0520 (9)	0.0378 (8)	0.0095 (7)	0.0002 (7)

### Geometric parameters (Å, °)

**Table:**

C1—C2	1.390 (3)	C11—C12	1.548 (2)
C1—C6	1.401 (3)	C11—C15	1.577 (2)
C1—C7	1.513 (2)	C12—O3	1.188 (2)
C2—C3	1.386 (3)	C12—O4	1.317 (2)
C2—H2	0.9300	C13—C14	1.437 (4)
C3—C4	1.375 (3)	C13—O4	1.466 (3)
C3—H3	0.9300	C13—H13A	0.9700
C4—C5	1.382 (3)	C13—H13B	0.9700
C4—H4	0.9300	C14—H14A	0.9600
C5—C6	1.387 (3)	C14—H14B	0.9600
C5—H5	0.9300	C14—H14C	0.9600
C6—C8	1.508 (3)	C15—N4	1.437 (2)
C7—N1	1.466 (2)	C15—N3	1.441 (3)
C7—H7A	0.9700	C15—C16	1.531 (3)
C7—H7B	0.9700	C16—O5	1.190 (2)
C8—N2	1.451 (2)	C16—O6	1.311 (2)
C8—H8A	0.9700	C17—O6	1.466 (3)
C8—H8B	0.9700	C17—C18	1.471 (4)
C9—O1	1.208 (2)	C17—H17A	0.9700
C9—N3	1.365 (3)	C17—H17B	0.9700
C9—N1	1.376 (2)	C18—H18A	0.9600
C10—O2	1.223 (2)	C18—H18B	0.9600
C10—N4	1.354 (3)	C18—H18C	0.9600
C10—N2	1.363 (2)	N3—H3A	0.87 (3)
C11—N1	1.440 (2)	N4—H4A	0.85 (3)
C11—N2	1.445 (2)		
			
C2—C1—C6	119.23 (17)	C14—C13—H13A	109.9
C2—C1—C7	119.00 (17)	O4—C13—H13A	109.9
C6—C1—C7	121.69 (16)	C14—C13—H13B	109.9
C3—C2—C1	121.0 (2)	O4—C13—H13B	109.9
C3—C2—H2	119.5	H13A—C13—H13B	108.3
C1—C2—H2	119.5	C13—C14—H14A	109.5
C4—C3—C2	119.5 (2)	C13—C14—H14B	109.5
C4—C3—H3	120.3	H14A—C14—H14B	109.5
C2—C3—H3	120.3	C13—C14—H14C	109.5
C3—C4—C5	120.1 (2)	H14A—C14—H14C	109.5
C3—C4—H4	120.0	H14B—C14—H14C	109.5
C5—C4—H4	120.0	N4—C15—N3	114.94 (17)
C4—C5—C6	121.16 (19)	N4—C15—C16	110.13 (16)
C4—C5—H5	119.4	N3—C15—C16	109.09 (16)
C6—C5—H5	119.4	N4—C15—C11	102.62 (15)
C5—C6—C1	118.94 (18)	N3—C15—C11	101.34 (14)
C5—C6—C8	119.12 (17)	C16—C15—C11	118.67 (15)
C1—C6—C8	121.91 (16)	O5—C16—O6	125.5 (2)
N1—C7—C1	115.68 (14)	O5—C16—C15	121.54 (18)
N1—C7—H7A	108.4	O6—C16—C15	112.92 (15)
C1—C7—H7A	108.4	O6—C17—C18	108.6 (3)
N1—C7—H7B	108.4	O6—C17—H17A	110.0
C1—C7—H7B	108.4	C18—C17—H17A	110.0
H7A—C7—H7B	107.4	O6—C17—H17B	110.0
N2—C8—C6	113.76 (15)	C18—C17—H17B	110.0
N2—C8—H8A	108.8	H17A—C17—H17B	108.3
C6—C8—H8A	108.8	C17—C18—H18A	109.5
N2—C8—H8B	108.8	C17—C18—H18B	109.5
C6—C8—H8B	108.8	H18A—C18—H18B	109.5
H8A—C8—H8B	107.7	C17—C18—H18C	109.5
O1—C9—N3	126.68 (18)	H18A—C18—H18C	109.5
O1—C9—N1	125.85 (18)	H18B—C18—H18C	109.5
N3—C9—N1	107.45 (16)	C9—N1—C11	111.93 (14)
O2—C10—N4	126.78 (16)	C9—N1—C7	120.52 (15)
O2—C10—N2	125.11 (18)	C11—N1—C7	121.64 (15)
N4—C10—N2	108.09 (16)	C10—N2—C11	113.25 (15)
N1—C11—N2	113.91 (14)	C10—N2—C8	124.21 (15)
N1—C11—C12	111.49 (13)	C11—N2—C8	122.46 (13)
N2—C11—C12	109.92 (14)	C9—N3—C15	114.32 (16)
N1—C11—C15	103.63 (14)	C9—N3—H3A	122.6 (16)
N2—C11—C15	101.80 (13)	C15—N3—H3A	122.3 (16)
C12—C11—C15	115.76 (14)	C10—N4—C15	113.12 (15)
O3—C12—O4	126.43 (17)	C10—N4—H4A	123.5 (17)
O3—C12—C11	124.36 (17)	C15—N4—H4A	123.0 (17)
O4—C12—C11	109.19 (14)	C12—O4—C13	116.92 (16)
C14—C13—O4	108.9 (2)	C16—O6—C17	116.28 (18)
			
C6—C1—C2—C3	−1.4 (3)	O1—C9—N1—C7	−17.8 (3)
C7—C1—C2—C3	175.22 (17)	N3—C9—N1—C7	163.56 (16)
C1—C2—C3—C4	0.2 (3)	N2—C11—N1—C9	97.72 (17)
C2—C3—C4—C5	1.3 (3)	C12—C11—N1—C9	−137.17 (15)
C3—C4—C5—C6	−1.5 (3)	C15—C11—N1—C9	−12.01 (18)
C4—C5—C6—C1	0.3 (3)	N2—C11—N1—C7	−55.3 (2)
C4—C5—C6—C8	−177.86 (17)	C12—C11—N1—C7	69.8 (2)
C2—C1—C6—C5	1.2 (2)	C15—C11—N1—C7	−165.00 (14)
C7—C1—C6—C5	−175.36 (16)	C1—C7—N1—C9	−78.3 (2)
C2—C1—C6—C8	179.26 (16)	C1—C7—N1—C11	72.4 (2)
C7—C1—C6—C8	2.7 (2)	O2—C10—N2—C11	−178.59 (18)
C2—C1—C7—N1	124.69 (18)	N4—C10—N2—C11	−0.2 (2)
C6—C1—C7—N1	−58.8 (2)	O2—C10—N2—C8	4.8 (3)
C5—C6—C8—N2	−126.25 (17)	N4—C10—N2—C8	−176.82 (17)
C1—C6—C8—N2	55.7 (2)	N1—C11—N2—C10	−116.92 (17)
N1—C11—C12—O3	−3.8 (3)	C12—C11—N2—C10	117.15 (16)
N2—C11—C12—O3	123.5 (2)	C15—C11—N2—C10	−6.07 (19)
C15—C11—C12—O3	−121.9 (2)	N1—C11—N2—C8	59.8 (2)
N1—C11—C12—O4	177.71 (15)	C12—C11—N2—C8	−66.1 (2)
N2—C11—C12—O4	−55.00 (19)	C15—C11—N2—C8	170.64 (15)
C15—C11—C12—O4	59.6 (2)	C6—C8—N2—C10	99.2 (2)
N1—C11—C15—N4	127.94 (15)	C6—C8—N2—C11	−77.2 (2)
N2—C11—C15—N4	9.48 (18)	O1—C9—N3—C15	177.73 (18)
C12—C11—C15—N4	−109.69 (17)	N1—C9—N3—C15	−3.7 (2)
N1—C11—C15—N3	8.88 (17)	N4—C15—N3—C9	−113.33 (19)
N2—C11—C15—N3	−109.58 (15)	C16—C15—N3—C9	122.44 (18)
C12—C11—C15—N3	131.26 (16)	C11—C15—N3—C9	−3.5 (2)
N1—C11—C15—C16	−110.44 (17)	O2—C10—N4—C15	−174.2 (2)
N2—C11—C15—C16	131.09 (17)	N2—C10—N4—C15	7.4 (2)
C12—C11—C15—C16	11.9 (2)	N3—C15—N4—C10	98.4 (2)
N4—C15—C16—O5	−29.5 (3)	C16—C15—N4—C10	−137.96 (18)
N3—C15—C16—O5	97.5 (2)	C11—C15—N4—C10	−10.7 (2)
C11—C15—C16—O5	−147.25 (19)	O3—C12—O4—C13	0.2 (3)
N4—C15—C16—O6	153.07 (17)	C11—C12—O4—C13	178.62 (19)
N3—C15—C16—O6	−79.91 (19)	C14—C13—O4—C12	150.2 (3)
C11—C15—C16—O6	35.3 (2)	O5—C16—O6—C17	2.3 (3)
O1—C9—N1—C11	−171.16 (18)	C15—C16—O6—C17	179.63 (19)
N3—C9—N1—C11	10.2 (2)	C18—C17—O6—C16	166.1 (2)

### Hydrogen-bond geometry (Å, °)

**Table:**

*D*—H···*A*	*D*—H	H···*A*	*D*···*A*	*D*—H···*A*
C7—H7A···O3	0.97	2.52	3.107 (2)	119
C17—H17B···O2^i^	0.97	2.58	3.343 (3)	136
C7—H7B···O3^ii^	0.97	2.59	3.478 (2)	153
N4—H4A···O2^iii^	0.85 (3)	2.05 (3)	2.871 (2)	165 (2)
N3—H3A···O5^iv^	0.87 (3)	2.09 (3)	2.917 (2)	159 (2)

Symmetry codes: (i) *x*−1, *y*, *z*; (ii) −*x*+1, −*y*+1, −*z*; (iii) −*x*+2, −*y*+1, −*z*+1; (iv) −*x*+1, −*y*+1, −*z*+1.
